# Evidence of tool use in a seabird

**DOI:** 10.1073/pnas.1918060117

**Published:** 2019-12-30

**Authors:** Annette L. Fayet, Erpur Snær Hansen, Dora Biro

**Affiliations:** ^a^Department of Zoology, University of Oxford, Oxford OX1 3PS, United Kingdom;; ^b^South Iceland Nature Research Centre, Ægisgata 2, 900 Vestmannaeyjar, Iceland

**Keywords:** tool use, seabird, animal cognition

## Abstract

Documenting novel cases of tool use in wild animals can inform our understanding of the evolutionary drivers of the behavior’s emergence in the natural world. We describe a previously unknown tool-use behavior for wild birds, so far only documented in the wild in primates and elephants. We observed 2 Atlantic puffins at their breeding colonies, one in Wales and the other in Iceland (the latter captured on camera), spontaneously using a small wooden stick to scratch their bodies. The importance of these observations is 3-fold. First, while to date only a single form of body-care-related tool use has been recorded in wild birds (anting), our finding shows that the wild avian tool-use repertoire is wider than previously thought and extends to contexts other than food extraction. Second, we expand the taxonomic breadth of tool use to include another group of birds, seabirds, and a different suborder (Lari). Third, our independent observations span a distance of more than 1,700 km, suggesting that occasional tool use may be widespread in this group, and that seabirds’ physical cognition may have been underestimated.

The evolution of tool use is one of the most enduring puzzles in behavioral biology. Investigating the distribution of tool use across taxa is key to understanding its adaptive value and hence its evolution in the natural world, and ultimately to understanding the evolutionary history of our own species. Tool use is defined as “the exertion of control over a freely manipulable external object (the tool) with the goal of (1) altering the physical properties of another object, substance, surface or medium (the target, which may be the tool user or another organism) via a dynamic mechanical interaction, or (2) mediating the flow of information between the tool user and the environment or other organisms in the environment” (1). Tools can be used for several purposes, mainly related to feeding, defense, aggression, social displays, or physical maintenance (2). “True” tool use requires manipulation of an object detached from the substrate, unlike “borderline” tool use where the tool remains part of the substrate (3). Tool use is a rare but phylogenetically widespread behavior in the wild. It is most common in birds and mammals, mainly in the Passeriformes and Primates orders (2), some of which use or even manufacture tools to complete complex tasks (4–6). Tool use appears to span a continuum between 2 broad types: “genetically based” behavioral specializations, inflexible and applied in a single context, and more flexible behavioral innovations, whose ontogeny may also rely partially on genetics but which can be applied creatively to new contexts (7). The ability of animals to use tools creatively has been related to their cognitive capacities (ref. 8,but see ref. 9).

Animal tool use is most frequent, and has been most discussed, in a need-for-resources framework, mainly related to feeding (10). Using tools for physical maintenance is also relatively common; for example, chimpanzees use tools to groom, scratch, or wipe themselves (11). In birds, captive parrots have been reported to scratch with sticks (2), but to date the only avian tool use for physical maintenance reported in the wild is “anting” (depositing ants on one’s plumage), observed in many species (mostly passerines) (10).

Here we provide evidence of a wild bird performing another form of tool use for physical maintenance. We observed 2 Atlantic puffins, *Fratercula arctica*, Charadriiform seabirds, scratching with a stick. We describe our observations and discuss their implications in the context of animal tool use.

## Methods

Puffins nest on colonies around the North Atlantic, mostly on grassy slopes on predator-free islands. As part of a study on Skomer Island, Wales (51°44′N; 5°19′W), observations have been made each June since 2012 at dusk, when puffins gather on the colony to preen, sleep, and socialize. We observed birds’ behavior with a spotting scope.

In July 2018, Browning motion-activated cameras were deployed near puffin nests on Grimsey Island, Iceland (66°32′N, 18°00′W), to record patterns of nest attendance. Cameras were configured to record 10 s of footage after each movement detection, with a minimum 30-s pause after each video.

### Data Availability.

All data are available as Movies S1–S3.

## Results

### Observation 1 (Wales).

On 18 June 2014 on Skomer Island an adult puffin was observed holding a wooden stick in its bill and using it to scratch its back for ∼5 s. The bird was sitting on the sea under the colony’s cliffs, among conspecifics. Shortly thereafter the bird took off (still holding the stick, albeit it is unclear for how long) and was lost from view.

### Observation 2 (Iceland).

**Fig. 1.**
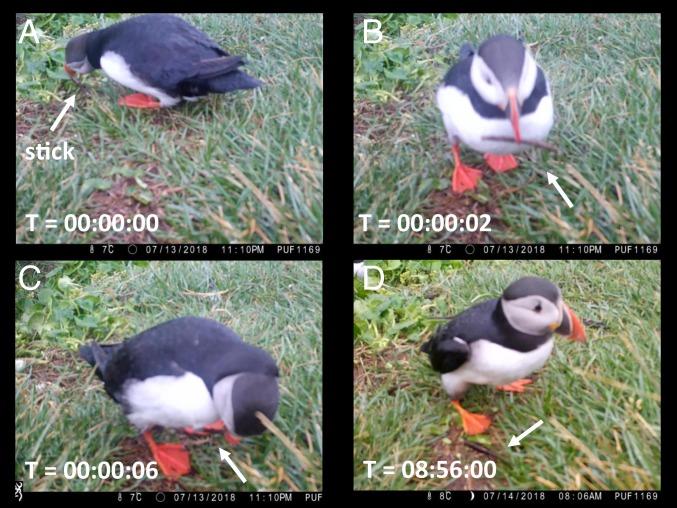
Screenshots of a puffin scratching with a stick. Time stamps (hours:minutes:seconds) indicate time elapsed since the first panel. The stick’s location is indicated by an arrow. (*A*) Puffin picking up the stick. (*B*) Puffin holding the stick. (*C*) Puffin scratching its chest with the stick. (*D*) Nine hours later, the stick is still visible on the ground.

On 13 July 2018 on Grimsey Island a camera trap recorded similar behavior (Movie S1). In the video, an adult puffin picks up a wooden stick from the ground (Fig. 1 *A*–*C*) then uses it to scratch its chest feathers (Fig. 1 *D* and *E*). The video stops shortly after this first bout of scratching. On later videos, the stick is on the ground (Fig. 1*F*). It eventually disappears after ∼24 h, likely displaced by a bird or the wind.

## Discussion

Our 2 instances of puffins using a stick as a tool for body care represent recorded evidence of a wild bird exhibiting this behavior, while to date, in the wild only primates and elephants have been observed scratching with a tool (2). It is also evidence of true tool use in a seabird (10), confirming the behavior in an avian order previously thought to lack the ability, need, or opportunity to use tools. Furthermore, our findings suggest that while this behavior is rare it is not restricted to a single population. Each of these conclusions has important implications for our understanding of the distribution and adaptive significance of tool use in the animal kingdom, and we discuss them in turn below.

Our observations of puffins rubbing their body with a stick fit the definition of tool use (1, 2, 10), as they involved the direct manipulation of a detached object toward a specific part of the environment (the birds’ plumage) with a specific goal (what this goal might be we discuss below). Before further discussion, however, it is important to note that our observations cannot be mistaken for the collection of nest material. Puffins preferentially collect soft material like grass or feathers to line their nests then quickly carry these inside their burrow, as frequently observed on both study colonies (Movies S2 and S3). In Wales, the puffin was sitting on the water and therefore was not collecting nest material on land. Puffins often assemble in rafts near the colony to rest, preen, and bathe. Our puffin engaged in body care like many of its neighbors and most likely picked up the stick on land before flying to the water. In Iceland, videos recorded after the tool-use episode showed the stick on the ground, confirming that the bird did not take it to its nest. We are therefore confident that our observed puffins did not pick up the sticks as nest-lining material.

Using sticks is common across tool-using taxa, but mostly in a foraging context to extract food from a cavity (5, 6). Our observations aside, stick tool use has exclusively been documented for extractive foraging in wild birds, which remains the primary use even in primates (10). Other, less common uses include communication or defense such as chimpanzee dominance displays (11), investigation of novel objects by captive New Caledonian crows (12), and scratching by primates, wild elephants, and captive parrots (2, 10). Since our observed puffins appeared to be rubbing the sticks on their plumage, it is reasonable to rule out foraging, investigation, or communication as the behavior’s function: Puffins only catch prey underwater, and they were not interacting with other puffins or probing objects with the stick. As such, they were most likely engaged in body care.

Two alternatives for the function of the stick can be proposed: It may have been used for its mechanical properties (e.g., to dislodge parasites or relieve an itch) or its chemical properties [in a manner similar to anting, where birds rub ants or plants on their plumage, presumably for their antiparasitic properties (13)]. The latter hypothesis seems less likely as the sticks used by our puffins seemed dry and therefore unlikely to have released chemical substances. As regards the former hypothesis, the absence, so far, of reports of wild birds using sticks as mechanical tools for preening could be due to a lack of need for this behavior, as birds can access most of their body with their beak. Nonetheless, reports of captive parrots scratching with stick-like objects suggest this behavior may exist in the wild but has remained unreported due to its rarity. The case of our puffins may reflect a specific ecological need which only occurs in some circumstances. For example, puffins suffer from seabird ticks, *Ixodes uriae*, which were particularly abundant on Grimsey Island in the summer of 2018. The stick may have helped with scratching or dislodging them, perhaps more effectively than the beak. In either case, mechanical or chemical application, investigating the role of parasites as potential drivers of the emergence of body-care-related tool use (e.g., by testing whether tool use prevalence correlates with parasite load in populations) would be an interesting avenue for future research.

Thus, our observations indicate that wild birds may have a wider tool-use repertoire for physical self-maintenance than current evidence suggests. The fact that several species of parrots showed this behavior in captivity further supports this hypothesis, and the pattern of such behavior having been observed multiple times independently and in different species may suggest that the behavior may not simply be an artifact of captivity. Furthermore, the similarity of tool use between birds and primates has been mainly discussed in the context of feeding (9); our findings highlight the need to broaden this discussion to include other functions such as physical maintenance.

More broadly, our findings provide evidence of true tool use in a seabird. This suggests tool use is rare in this group, but can no longer be considered absent. Tool use is present in a small number of species [less than 1% of known genera (14)] and is mostly related to feeding, presumably because of the high fitness gains reaped by accessing concealed food sources, especially when these are more profitable than nonconcealed ones (15). Seabirds feed at sea, mainly on fish, and have evolved unparalleled abilities to dive, swim, and catch prey underwater. The ocean seems an unlikely setting for seabirds to evolve tool use, not least because of the lack of objects to use as tools and of concealed food sources in the water. Tool use, indeed, seems even rarer in aquatic animals than terrestrial ones (16). Seabirds only visit land to breed, which limits the opportunity for tool use and could favor its use for nonforaging purposes like courtship or physical maintenance. Such behaviors may also remain unreported because seabirds are difficult to observe: They spend most of their time at sea, underground, or on inaccessible cliffs, and many are nocturnal. Our finding of another physical-maintenance tool-use behavior in wild birds besides anting suggests that tool use can emerge without strong selective pressure to obtain resources.

The fact that our 2 observations occurred on distant populations also raises interesting questions regarding their implications for the birds’ underlying cognition. One possibility is that the behavior arose by independent behavioral innovations as flexible problem solving by the puffins observed, or that they socially learned this behavior from other innovators. Alternatively, the behavior could have a genetic component (in that it appears along a fixed developmental pathway during ontogeny, without the need for innovation), present in both populations but rarely exhibited. Currently we cannot distinguish between these scenarios; careful experimentation and information on the birds’ history of interactions with sticks and conspecifics may reveal the extent to which stick use represents behavioral innovation and has the potential for social transmission. The propensity for behavioral innovation has been shown to increase with relative brain size in birds and primates (8). Seabirds’ relative brain size is comparatively small (17) and they are not generally described as possessing sophisticated cognitive abilities. However, they feed in patchy, unpredictable environments, where they must integrate multiple sources of physical and social information to make complex decisions in space and time. Solving such problems requires behavioral flexibility and skills in multiple domains including learning, memory, and planning, also evidenced by high levels of fidelity in migration and foraging routes in numerous species (18, 19). As such, seabirds’ cognitive capacities may have been considerably underestimated. The fact that to date the only other birds seen scratching with a stick are parrots, prolific tool users and problem solvers (20), supports this hypothesis.

In sum, our discovery of another type of tool use in wild birds, outside of the Passeriform order where most avian tool use is known to occur, and of a form so far restricted in the wild to primates and elephants, highlights the importance of widening the discussion on the evolution of animal tool use to a broader framework. While efforts to identify a single unifying driver for the emergence of all tool use are unlikely to succeed, a more complete picture of the range of contexts and taxa in which tool use occurs will allow us to break the phenomenon down into more meaningful categories for analysis. We therefore encourage researchers to include species not traditionally considered as good candidates for tool use and to report unusual behaviors across species. Our finding also warrants further studies on seabird cognition, a topic almost entirely unstudied but clearly rife with opportunity for future research.

## Supplementary Material

Supplementary File

Supplementary File

Supplementary File
